# Genome wide interactions of wild-type and activator bypass forms of σ54

**DOI:** 10.1093/nar/gkv597

**Published:** 2015-06-16

**Authors:** Jorrit Schaefer, Christoph Engl, Nan Zhang, Edward Lawton, Martin Buck

**Affiliations:** 1 Faculty of Natural Sciences, Division of Cell & Molecular Biology, Imperial College London, London SW7 2AZ, UK; 2 Institute for Global Food Security, Queen's University Belfast, Belfast BT9 5BN, UK

## Abstract

Enhancer-dependent transcription involving the promoter specificity factor σ^54^ is widely distributed amongst bacteria and commonly associated with cell envelope function. For transcription initiation, σ^54^-RNA polymerase yields open promoter complexes through its remodelling by cognate AAA+ ATPase activators. Since activators can be bypassed *in vitro*, bypass transcription *in vivo* could be a source of emergent gene expression along evolutionary pathways yielding new control networks and transcription patterns. At a single test promoter *in vivo* bypass transcription was not observed. We now use genome-wide transcription profiling, genome-wide mutagenesis and gene over-expression strategies in *Escherichia coli*, to (i) scope the range of bypass transcription *in vivo* and (ii) identify genes which might alter bypass transcription *in vivo*. We find little evidence for pervasive bypass transcription *in vivo* with only a small subset of σ^54^ promoters functioning without activators. Results also suggest no one gene limits bypass transcription *in vivo*, arguing bypass transcription is strongly kept in check. Promoter sequences subject to repression by σ^54^ were evident, indicating loss of *rpoN* (encoding σ^54^) rather than creating *rpoN* bypass alleles would be one evolutionary route for new gene expression patterns. Finally, cold-shock promoters showed unusual σ^54^-dependence *in vivo* not readily correlated with conventional σ^54^ binding-sites.

## INTRODUCTION

Amongst the strategies used to regulate transcription initiation in bacteria, the emergence of the enhancer-dependent system that uses the σ^54^ RNA polymerase is of special interest because it deviates from the transcription factor recruitment mechanisms commonly seen (1,2). Rather, binding and hydrolysis of aenosine triphosphate (ATP) by cognate AAA+ ATPase activator proteins remodel σ^54^ containing closed promoter complexes to yield open promoter complexes (3,4). Potentially, σ^54^ initially acted as a global repressor of RNA polymerase (RNAP) activity since it effectively road blocks the DNA entry path used for delivering melted DNA into the DNA binding cleft of RNAP. This silenced state of RNAP may then have become a target for regulation. Intriguingly, to date no bacteria have been found where the gene encoding σ^54^ (*rpoN*) is present in the absence of any cognate AAA+ activator (4,5). This suggests forms of σ^54^ functioning without activators are uncommon. Yet variants of σ^54^ bypassing activator requirements for making open promoter complexes exist and have been characterized extensively *in vitro* (6,7). Single amino acid substitutions in σ^54^ can yield activator bypass forms. Quite why σ^54^ has seemingly not (or rarely) evolved to activator independence is rather unclear. Here we have investigated the *in vivo* global actions of wild-type σ^54^ in *Escherichia coli* bacteria and one bypass variant to explore potential restrictions on evolving bypass gene regulation systems.

We found that only a very limited number of knock-outs (KOs) of non-essential genes in *E. coli* increased activator bypass transcription suggesting multifactorial global constraints prevent unregulated transcription by the σ^54^ containing RNAP holoenzyme (σ^54^RNAP). Intriguingly, some genes with σ^54^ binding sites were found to be repressed in a σ^54^-dependent manner. In some cases the putative σ^54^ binding site overlapped that of a putative σ^70^ binding site suggesting a competition for promoter sites, which we confirmed by *in vitro* experiments. In other cases, σ^54^ and σ^70^ RNAP appear to act antagonistically through e.g. convergent transcription events.

## MATERIALS AND METHODS

### Bacterial strains and growth conditions

For the construction of the *gln*A_p2_ strains (Supplementary Table S1) P1*_vir_* transduction was used to insert *rpoN208*::Tn10 (K1471 donor strain) into the native chromosomal *rpoN* locus of BW25113 (recipient). Subsequently, transformation was used to transfer the pSB4A3 plasmid harbouring ΦP*_gln_*_Ap2_-*gfp* (a gift from Baojun Wang, University of Edinburgh, UK) and pBAD18 cm (containing *Klebsiella pneumonia rpoN, rpoN_ΔRI_* or vector only). For the construction of the *pspA* strains (Supplementary Table S1) P1*_vir_* transduction was used to insert *rpoN208*::Tn10 (K1471 donor strain) into the native chromosomal *rpoN* locus of BW25113 (recipient). Next the ΦP*_pspA_*-*lacZ* (MVA4 donor strain; 8) was inserted into the *att* site of the BW25113 *rpoN208*::Tn10 chromosome, using P1*_vir_* transduction. Finally, transformation was used to transfer pBAD18cm (containing *Klebsiella pneumonia rpoN, rpoN_ΔRI_* or vector only) into the BW25113 *rpoN208*::Tn10 ΦP*_pspA_*-*lacZ*. The F3K3 plasmid containing the filamentous phage pIV secretin was also transformed into this strain. To transfer the single-gene KOs from the KEIO collection (9) and small peptide/ small RNA library (10), P1*_vir_* lysates were produced from the pooled strains. These were then used as a donor for P1*_vir_* transduction of the KOs into the *pspA*-SABRS recipient strain (Supplementary Table S1).

For gene expression assays *in vivo*, strains were grown on LB indicator (XGal and MacConkey) plates and in minimal Gutnick liquid media supplemented with L-glutamine (11) as the nitrogen source to allow good growth in the absence of σ^54^ function (since the nitrogen assimilation pathways in *E. coli* rely on σ^54^ for expressing appropriate levels of glutamine synthetase). The antibiotic concentrations used were 30 μg/ml kanamycin, 50 μg/ml chloramphenicol and 50–100 μg/ml ampicillin.

### Gene expression assays *invivo*

To measure activity of ΦP*_pspA_*-*lacZ in vivo*, β-Galactosidase assays were carried out as described (12). To determine gene expression levels of the ΦP*_gln_*_Ap2_-*gfp* reporter *in vivo*, cells were grown in black 96-well clear-bottom tissue culture plates. Optical density at 600 nm (OD_600_) and green fluorescence (excitation: 485 nm; emission: 520 ± 10 nm, gain: 1000) were measured simultaneously using a BMG FLUOstar Omega microplate reader. Promoter activity was expressed as fluorescence emission at 520 nm per OD_600_ measured in triplicate.

### Inverse PCR

Inverse polymerase chain reaction (PCR) was used to identify selected KO mutants (Supplementary Table S2). Chromosomal DNA from strains that were positive for β-galactosidase activity on indicator plates and in liquid culture (Miller assay) was purified and digested with *Nco*I, a 6-base cutter. Re-ligation of the resulting fragments into circular DNA was done in a volume of 100 μl. This DNA was used as a template for PCR, using kanamycin cassette-specific forward and reverse primers (9). Products were run on a gel, extracted and sequenced. NCBI Blast (http://blast.ncbi.nlm.nih.gov/Blast.cgi) was used to align sequenced regions to the *E. coli* chromosome and to determine the identity of the deleted gene.

### RNA sequencing (RNAseq)

A total of 100 ml cell cultures were grown to exponential phase at 42 or 37°C, in minimal Gutnick media supplemented with 5 mM L-Glutamine and 0.4% glucose. Cell pellets were sent to Vertis Biotechnologie AG (Germany) for further processing. Total RNA was isolated from the cell pellets using a bead mill and the mirVana RNA isolation kit (Ambion) including DNase treatment. Ribosomal RNA molecules were depleted from the total RNA preparations using the RiboZero rRNA Removal Kit (Bacteria) (Epicentre). The rRNA depleted RNAs were fragmented with RNase III. Then, the RNA fragments were poly(A)-tailed using poly(A) polymerase and the 5′ PPP structures were removed using RNA 5′ polyphosphatase (Epicentre). An RNA adapter was ligated to the 5′-phosphate of the RNA fragments. First-strand cDNA synthesis was performed using an oligo(dT)-adapter primer and M-MLV reverse transcriptase. The resulting cDNA was PCR-amplified using a high fidelity DNA polymerase. The cDNA was purified using the Agencourt AMPure XP kit (Beckman Coulter Genomics). The cDNA pools were size fractionated in the size range of 200–450 bp using a differential clean-up with the Agencourt AMPure kit. The primers used for PCR amplification were designed for TruSeq sequencing according to the instructions of Illumina and sequenced on a Illumina HiSeq 2000 system using 50 bp read length. Near equimolar amounts of cDNA were used for sequencing. The cDNA reads were analysed via the RNA-seq workflow within Partek^®^ Genomics suite 6.6, including a QA/QC step to gauge the sequencing quality. Each sample yielded close to equivalent total reads with 100% of reads aligned to the *E. coli* K-12 reference genome (NC_000913) (Supplementary Table S4). For each gene, reads were normalized for both the number of reads and length of RNA and expressed as RPKM (reads per kilobase per million) (13; Supplementary Table S4). A one-way ANOVA statistical analysis was performed to test for significant differences in genome-wide gene expression between all 3 strains (Supplementary Table S4).

### *In* *vitro* spRNA assays

Small primed RNAs (spRNAs) were synthesized in 10 μl volumes containing: 0.125 μM σ^70^/σ^54^RNAP holoenzyme, 20 nM linear promoters, 0.5 mM dinucleotide primers, 0.2 mg/ml heparin and 0.2 μCi/μl ^32^P-NTP in STA buffer (2.5 mM Tris-acetate pH8, 8 mM Mg-acetate, 10 mM KCl, 1 mM DTT, 3.5% (w/v) PEG 8000) at 37°C for 10 min. For the σ^70^/σ^54^ competition assays, 0.4 μM σ^54^ were either added before or with σ^70^ for binding competition. The reactions were quenched by formamide stop dye and run on a 20% sequencing gel. Signals were detected by Fuji PhosphorImager and quantified using the AIDA Image Analyzer software (raytest).

## RESULTS

### Genome-wide screens for increased bypass transcription suggest constraints exist

**Figure 1.**
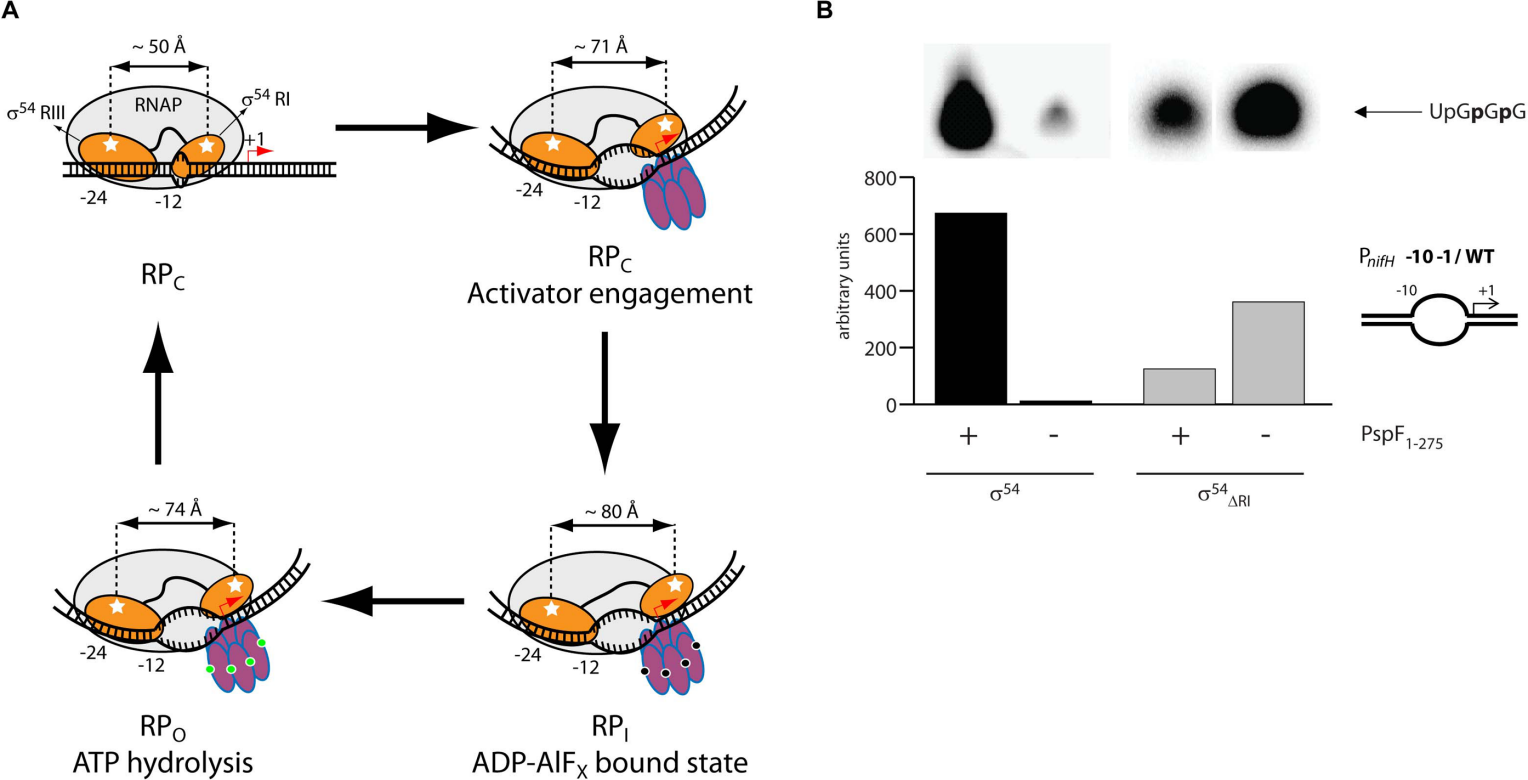
(**A**) The Region I of σ^54^ acts to inhibit RNA polymerase activity by blocking promoter DNA entry into the major DNA binding cleft. (**B**) Removal of Region I enables *in vitro* transcription from the late-melted *nifH* promoter (−10–1/WT) without the AAA+ activator PspF_1–275_.

**Figure 2.**
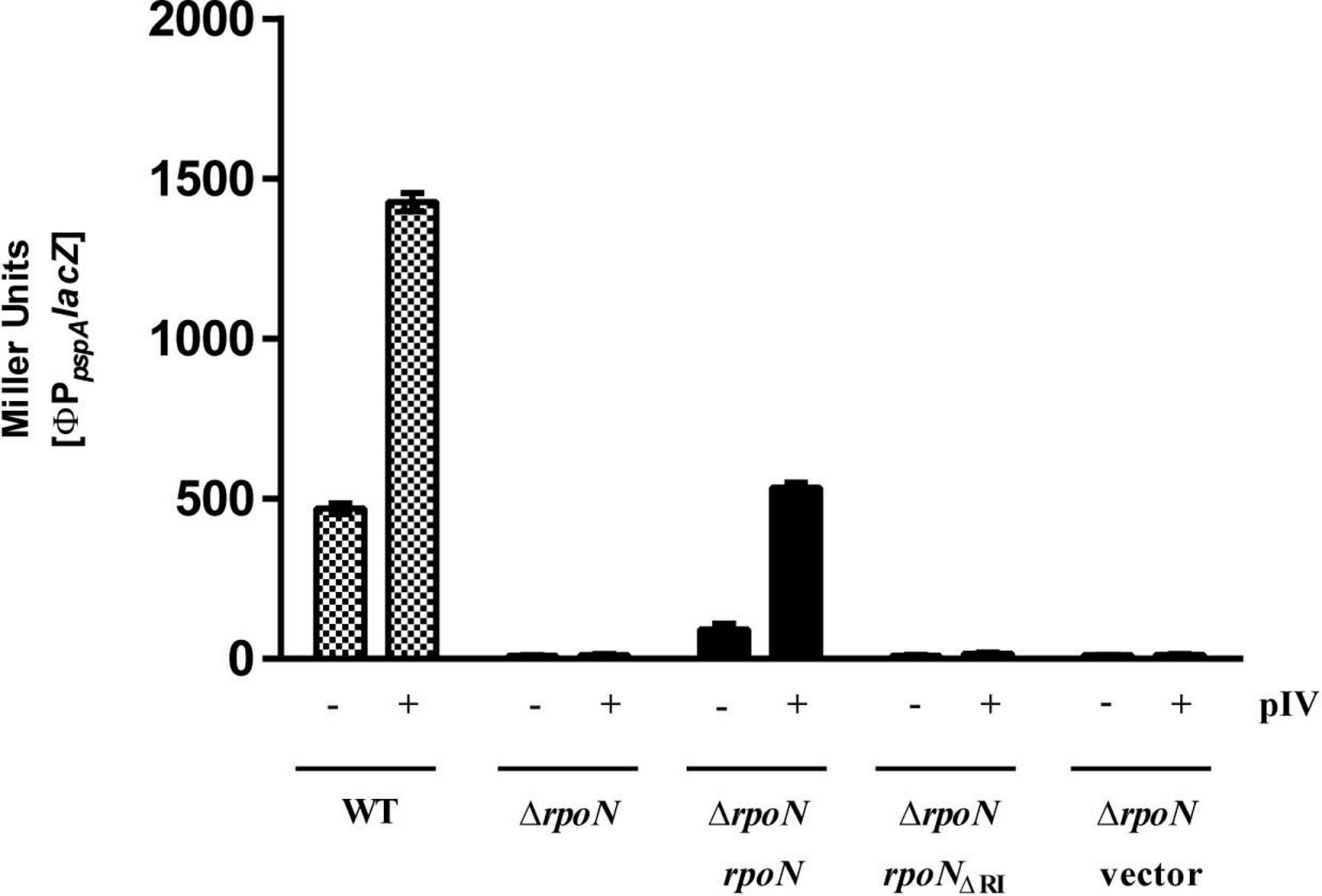
The σ^54^ dependent *pspA* promoter is active *in vivo* in the presence of stress inducing signals (here pIV secretin) and non-bypass wild-type σ^54^, but is inactive with the ΔRI σ^54^ form. Experiments were performed with three biological replicates.

A form of σ^54^ lacking the repressive Region I, σ^54^ΔRI, can bypass activator requirement for transcription *in vitro* (Figure 1;^14^,^15^). Using a *lacZ* fusion to the *pspA* promoter (P*_pspA_lacZ*) in single copy integrated into the *att* site of the *E. coli* chromosome we sought bypass activity from the σ^54^-dependent *pspA* promoter *in vivo*. Reporter strains lacked *rpoN* and were complemented with either the wild-type or the ΔRI form of σ^54^ expressed from pBAD18 cm (Supplementary Table S1, Figure S1). While *rpoN* and *rpoN_ΔRI_* were under control of the *araBAD* arabinose inducible promoter, leaky expression (without arabinose) yielded sufficient levels of σ^54^ to enable pIV-dependent transcription from the *pspA* promoter (Supplementary Figure S2). Thus unless stated otherwise (Supplementary Table S2) the experiments were performed in the absence of arabinose. Under standard growth conditions, no clear bypass transcription was observable with the σ^54^ΔRI form (Figure 2). Secretin pIV-dependent activation of the *pspA* promoter (16) via its cognate activator PspF acting on the σ^54^RNAP holoenzyme was clearly observed *in vivo* (Figure 2).

It was reported that increased temperature enhances σ^54^ activator bypass transcription *in vitro* at the *glnA*p2 promoter (6). We therefore cultured cells at 30, 37 and 42°C to favour detecting bypass transcription *in vivo*. Indeed, we observed a four-fold increase in σ^54^ΔRI-dependent *glnA*p2 promoter activity at 42°C compared to 30 or 37°C. This bypass activity accounts for almost 50% of wild-type σ^54^-dependent transcription (Figure 4).

To seek genetic determinants potentially restricting *in vivo* bypass transcription from the *pspA* promoter we introduced non-essential gene KOs into the reporter strains from the KEIO collection of *E. coli* gene KOs (9; a gift from the Carol Gross laboratory, UCSF, USA) and the small RNA/small peptide KO collection (10; a gift from the Gigi Storz laboratory, NIH, USA). Gene KOs were introduced into the reporter strains by phage P1*_vir_* transduction following growth of the phage on pooled KOs. Transductants were screened on indicator media (X-gal, MacConkey) or on lactose as a carbon source for increased expression of P*_pspA_lacZ*. Screens were carried out at 37 and 42°C, and in the absence and presence of 0.001% arabinose (Supplementary Table S2). Colony PCR was used to confirm all necessary genetic elements (*rpoN* KO, pBAD18 cm, *rpoN*ΔRI allele, P*_pspA_lacZ* fusion) were intact in candidate upregulated clones. Coverage was three to four-fold of the chromosome assuming all KOs were represented in the library.

**Figure 3.**
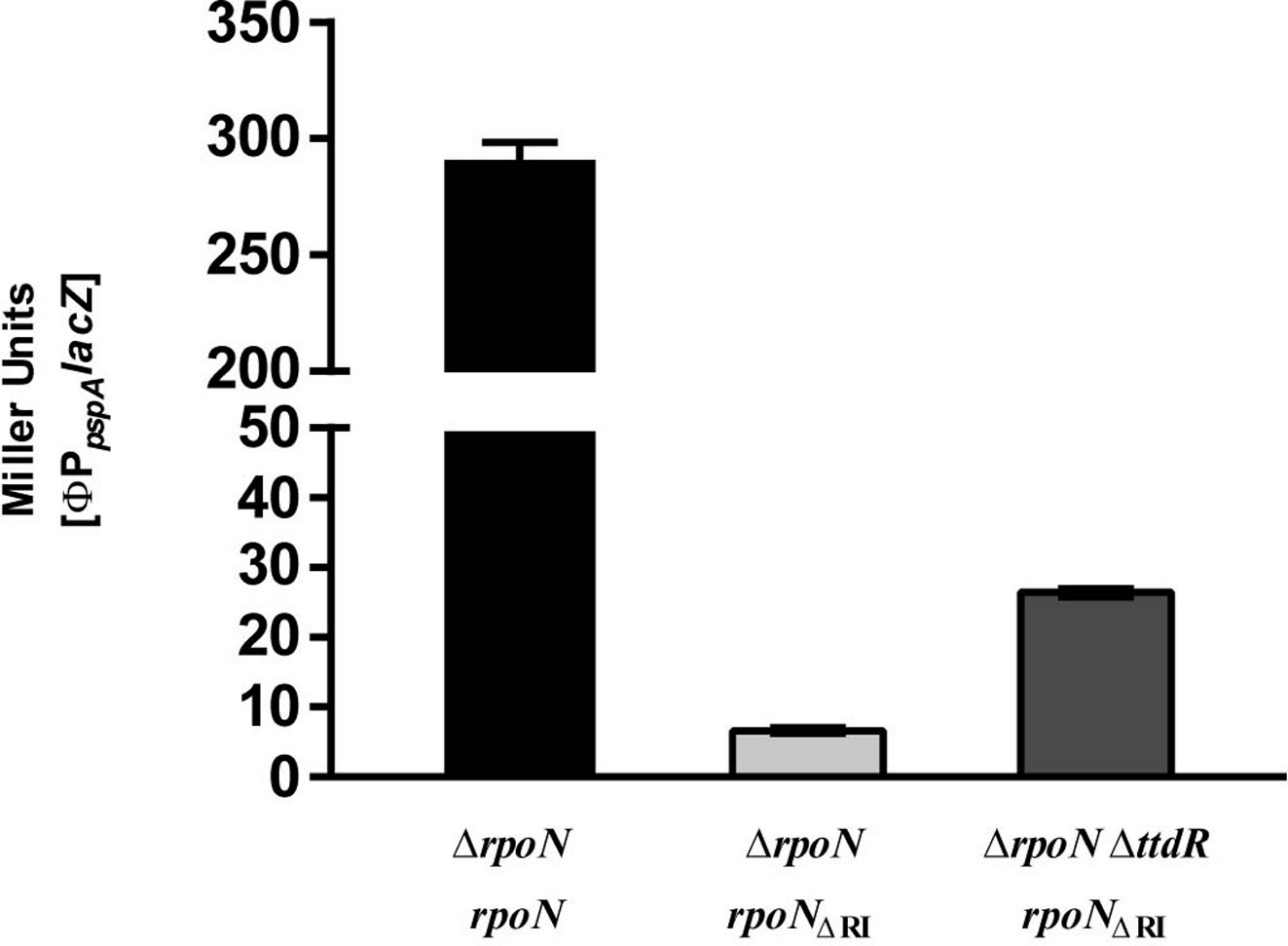
Single-gene knock-out screening at 37°C yielded *ttdR* (encoding a nucleoid DNA-binding protein) as negatively impacting σ^54^ bypass transcription from the *pspA* promoter *in vivo*. In addition to increased bypass transcription from the ΦP_pspA_*lacZ* on XGal and MacConkey screens (Supplementary Table S2), the Δ*ttdR* mutant also increased bypass transcription as measured in Miller Units. Experiments were performed with three biological replicates.

From the above screens, we found 26 KOs which led to apparent bypass transcription from the *pspA* promoter based on an increased β-galactosidase activity on indicator plates (Supplementary Table S2). Of these, six KOs (*asnA*, *greA*, *hldE*, *nanT*, *ttdR*, *yhbX*) which were found in a screen at 37°C also show increased β-galactosidase activity in liquid culture. These KOs were directly identified by inverse PCR and sequencing. Five KOs were discounted as being direct restrictive determinants of *in vivo* levels of bypass transcription on the basis of (i) just causing changes to the cell's envelope which led to enhanced β-galactosidase activity, potentially a permeability issue with the ONPG substrate or (ii) reconstitution of the native chromosomal *rpoN* locus. The *ttdR gene* however encodes a nucleoid binding protein and its loss seems to genuinely cause increased bypass transcription (Figure 3). The basis of the effect may lie in changes to DNA superhelicity, which is known to impact on bypass transcription *in vitro* (6). We conclude that in *E. coli* very few non-essential genes act to restrict bypass transcription *in vivo*. Those that do may act globally on e.g. DNA superhelicity and so may also impact on many additional gene expression events which themselves are not directly σ^54^ dependent.

### Overexpressed genes do not yield increased bypass transcription

We considered that the amount of a gene product might be limiting, rather than inhibitory, for supporting bypass transcription by σ^54^. We therefore introduced a library of genes from the closely related *Salmonella typhimurium* bacteria on a multi-copy plasmid (a gift from a gift from Diarmaid Hughes; Uppsala University, Sweden; 17) into the *E. coli* reporter strain and screened for increased expression of P*_pspA_lacZ*. None was observed, suggesting no single gene product limits bypass transcription in *E. coli* (Supplementary Table S2).

### RNAseq analysis of the σ^54^ bypass allele ΔRI activity reveals activator independent promoter sites

**Figure 4.**
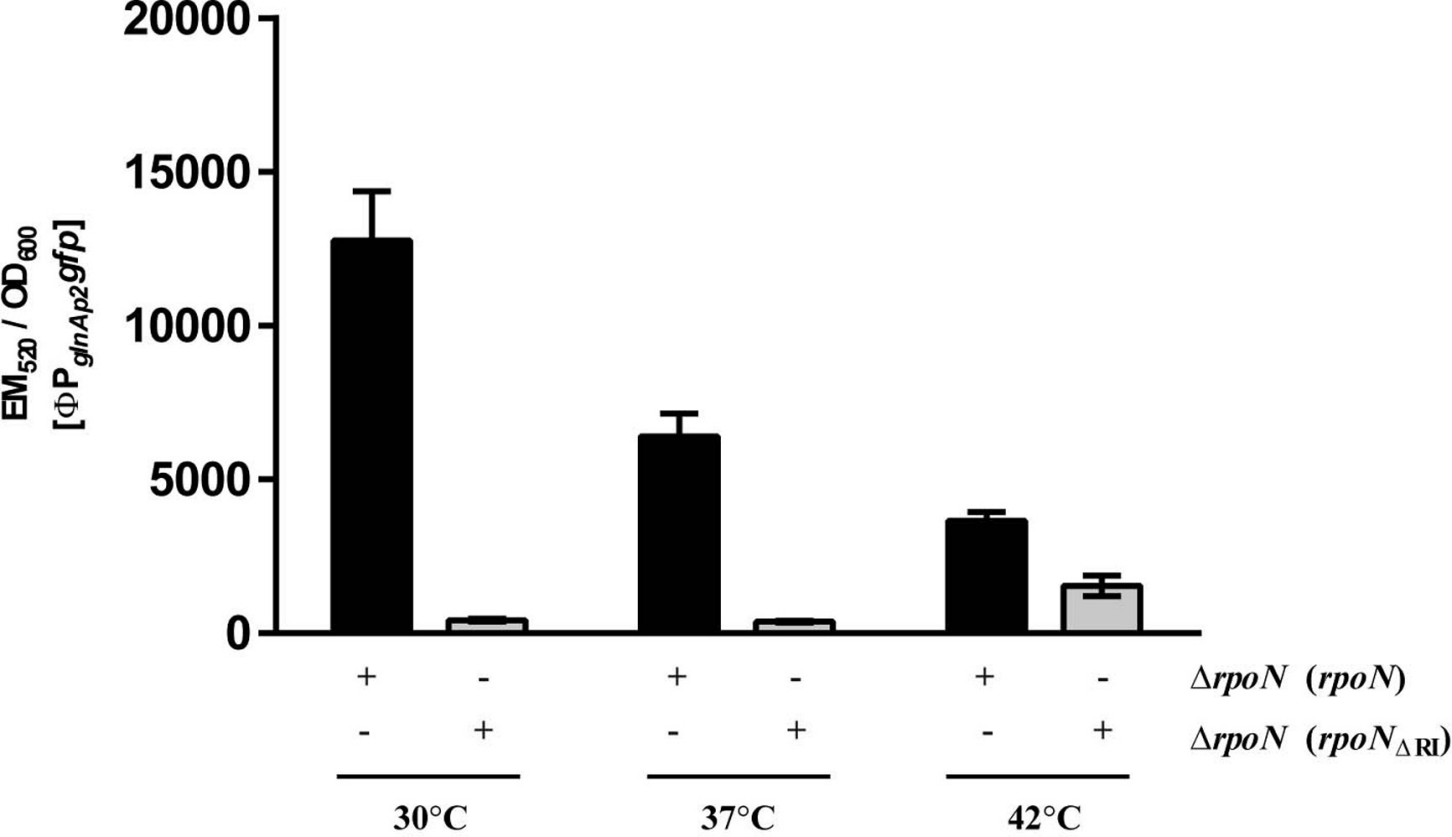
Elevated temperature increases bypass transcription from the *glnA*p2 promoter *in vivo* (grey), by over 40% activity of the wild-type *rpoN* control at the same temperature (black). Experiments were performed with three biological replicates.

We conducted RNAseq of cells containing the ΔRI bypass form of σ^54^ and compared outcomes to those from cells with wild-type σ^54^ and to cells lacking σ^54^. Cells were cultured at 42°C, which enhances bypass *in vitro* (6), to favour detecting bypass transcription *in vivo*. We confirmed using the *glnA*p2 promoter this was so *in vivo* (Figure 4). We chose minimal media supplemented with L-glutamine as the nitrogen source to allow good growth in the absence of σ^54^ function (since the nitrogen assimilation pathways in *E. coli* rely on σ^54^ for expressing appropriate levels of glutamine synthetase).

**Figure 5.**
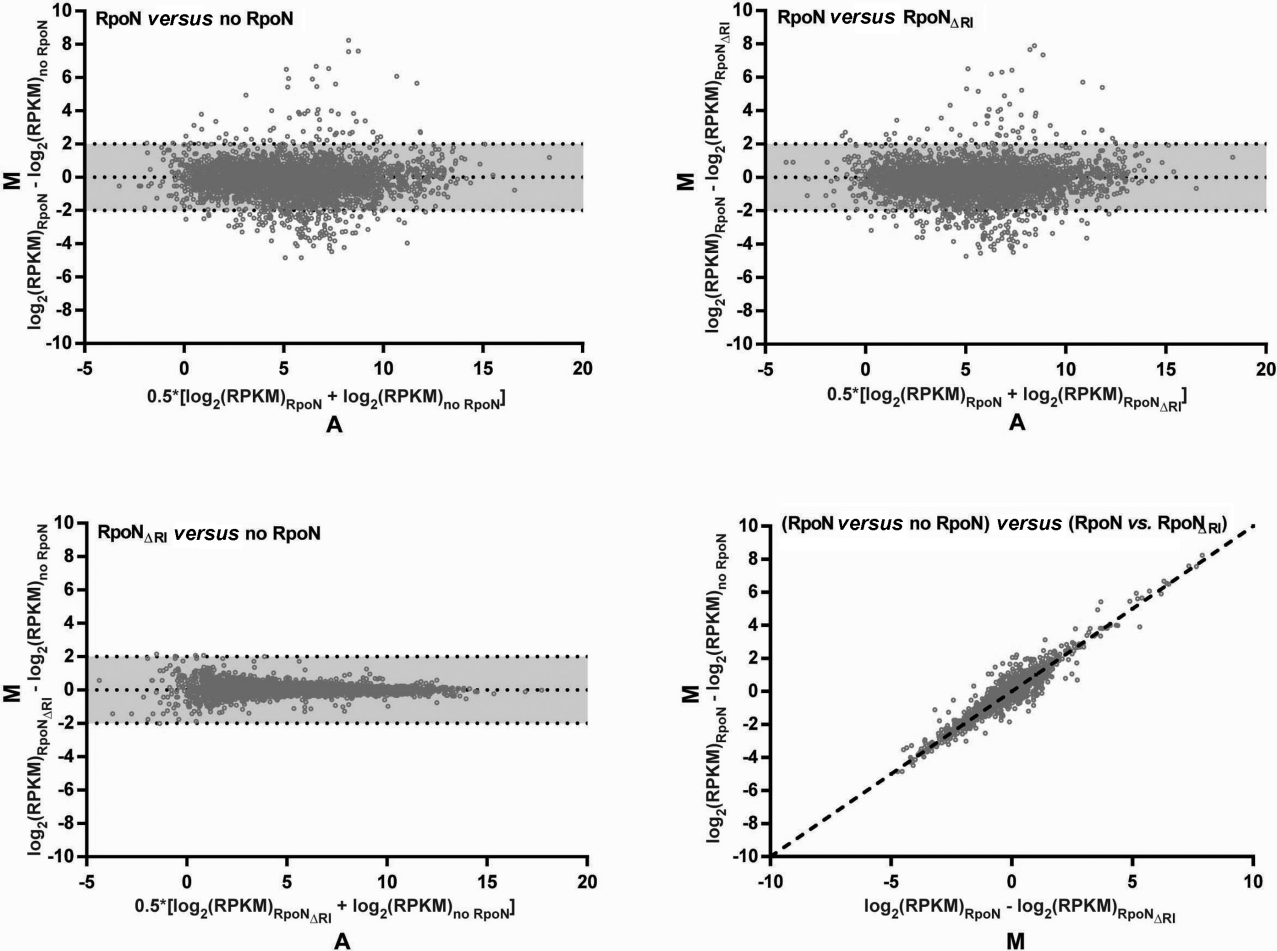
Genome-wide transcriptional profiling by RNAseq revealed no major global bypass transcription by RpoN_ΔRI_. Shown are MA scatterplots comparing cells with wild-type RpoN to cells with no RpoN (Top left) or RpoN_ΔRI_ (Top right) as well as cells with RpoN_ΔRI_ to cells with no RpoN (Bottom left). M is the difference in number of reads for each gene between a pair of strains expressed as log_2_(RPKM)_Strain 1_ – log_2_(RPKM)_Strain 2_, i.e. values > 0 indicate higher expression in strain 1 while values < 0 indicate higher expression in strain 2. A is the average number of reads of a gene between both strains expressed as 0.5*[log_2_(RPKM)_Strain 1_ + log_2_(RPKM)_Strain 2_]. Data points within the grey shaded area (*M* = log_2_(RPKM) = +/− 2) depict genes less than equal to four-fold differently expressed between the strains. The scatterplot in the Bottom right corner compares the *M*-values of the Top left and Top right plot showing that the differences in gene expression between cells with wild-type RpoN and no RpoN are similar to the differences in gene expression between cells with wildtype RpoN and RpoN_ΔRI_ (dotted line: y = x). The data is derived from single sample analysis.

RNAseq profiles of cells with wild-type σ^54^ compared to cells with σ^54^ΔRI or no σ^54^ revealed no major global bypass transcription (Figure 5). By scoring for more RNAseq reads in presence of the bypass σ^54^ΔRI form compared to the no σ^54^ we identified eight sites of low-level activator-independent transcription from sequences downstream of a canonical −12/−24 σ^54^ promoter DNA recognition sites (Table 1). We conclude that a small subset of σ^54^ binding sites can yield transcripts independently of the conventional AAA+ ATPase driven activation pathway, and that this bypassing is mediated by the loss of Region I of σ^54^. The C residue in the GC −12 element is conserved in 96% of bacterial σ^54^ promoters (18). Alignment (19,20) of the bypassing −12/−24 σ^54^ motifs indicates that the C residue is not strictly conserved at these promoters (Table 1). This finding is in line with *in vitro* studies showing that the consensus C at −12 is important for keeping basal transcription in check (21).

**Table 1.** (**A**) σ^54^ promoter sites showing increased bypass transcription at 42°C *in vivo* when Region I of σ^54^ is absent

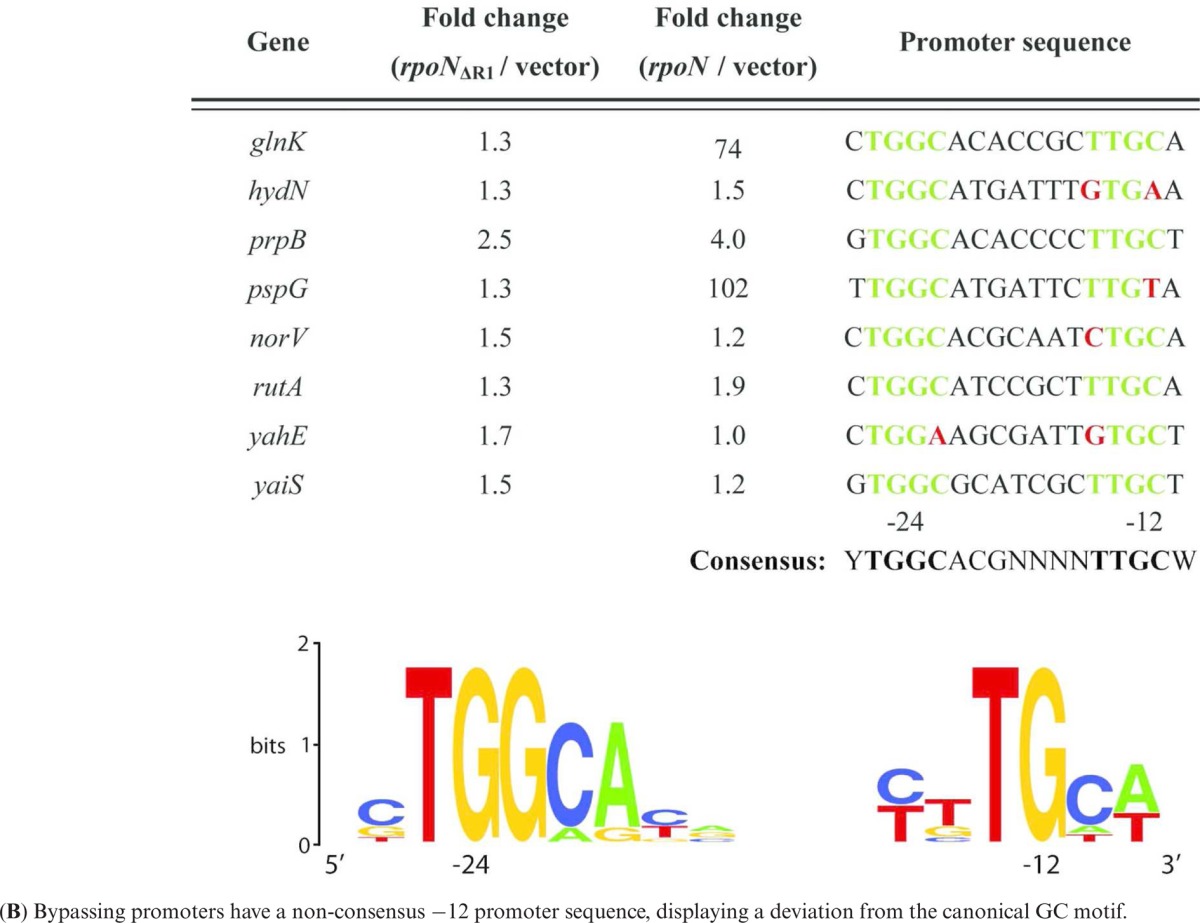

By scoring for more RNAseq reads in presence of wild-type σ^54^ compared to σ^54^ΔRI we identified canonical −12/−24 promoters that are likely to be activated in a Region I-dependent manner under the growth conditions of the experiment. Table 2 lists these promoters and the genes under their control. The majority are promoters from several well characterized σ^54^-dependent systems such as those controlled by the activators such as NtrC, PspF, PrpR and RtcR.

**Table 2.** σ^54^ promoter sites active at 42°C *in vivo*

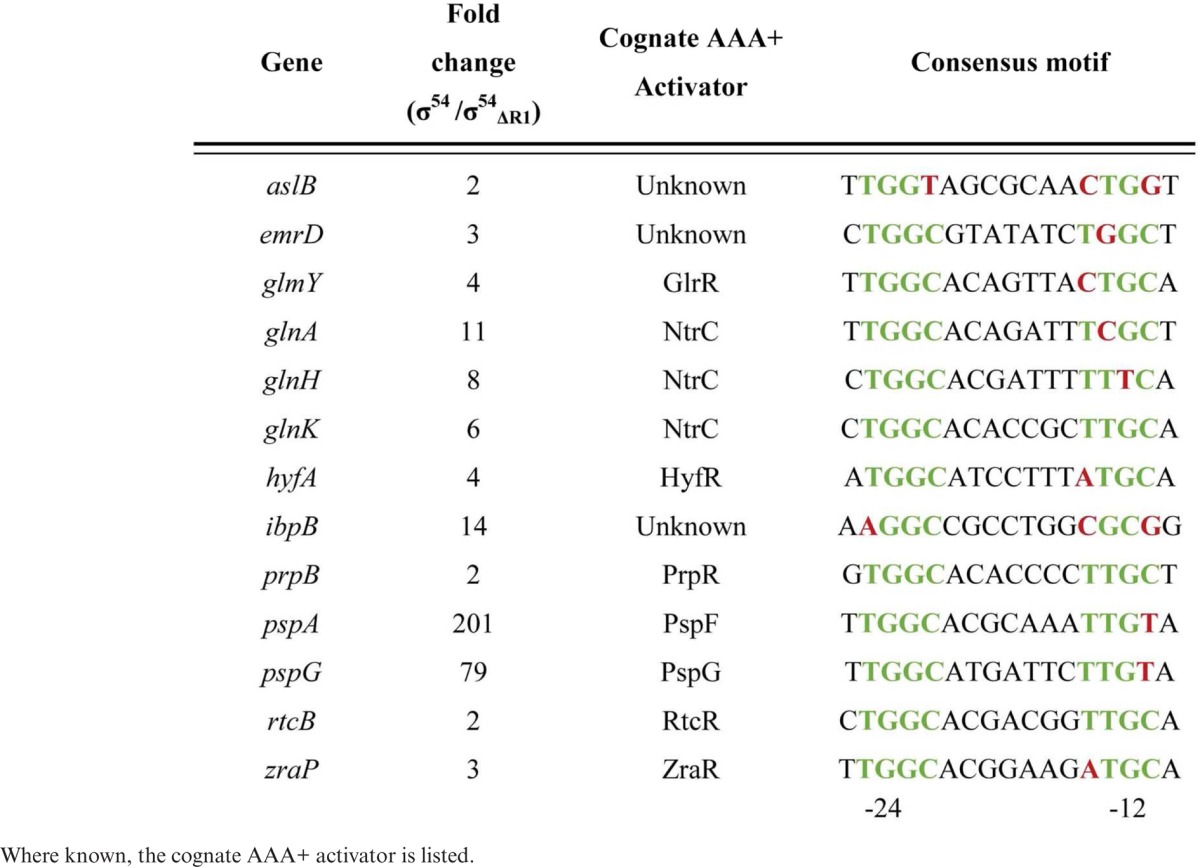

### σ^54^ is repressive at some promoters

**Figure 6.**
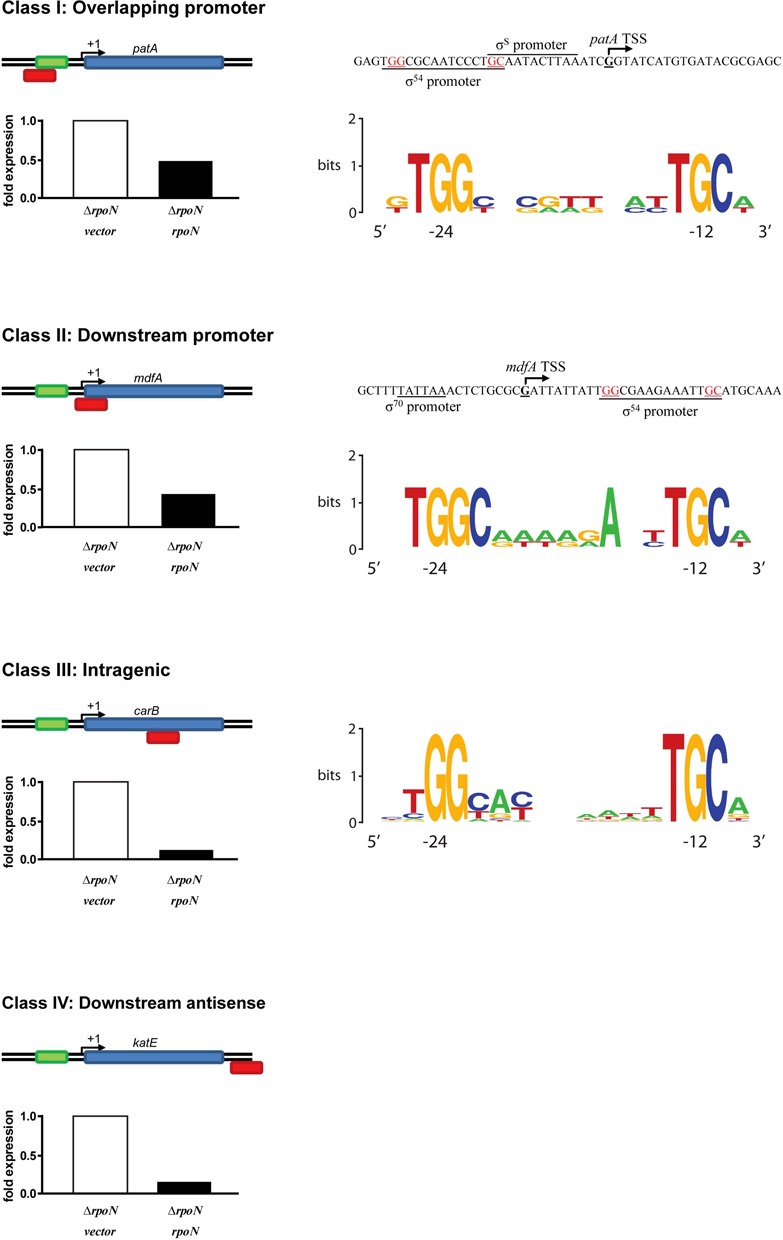
σ^54^ -dependent repression was observed at multiple genes with σ^54^ binding sites identified through ChIP Seq (Supplementary Table S3). These genes were grouped in 4 classes based on the location of the σ^54^ motifs relative to the promoter sequences. Shown are examples of each class and consensus motifs of class I–III. Class IV consists of only one member, *katE*.

**Figure 7.**
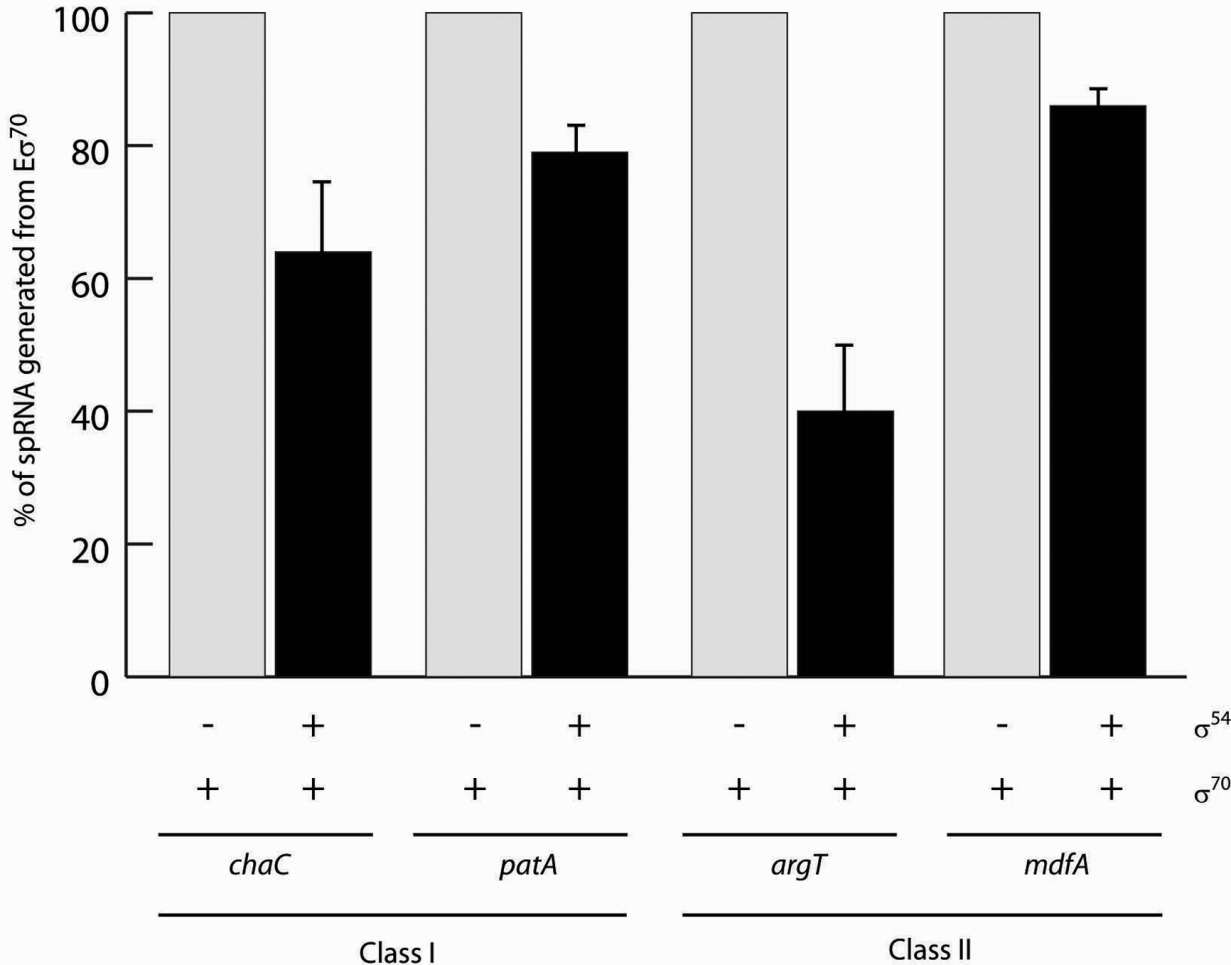
σ^54^ represses transcription by σ^70^*in vitro*. Purified holoenzymes were incubated with linear homoduplexes from four promoters (*argT, chaC, mdfA* and *patA* WT/WT) and transcription was measured using dinucleotide primers (−1/+1 or +1/+2) and a radioactive rNTP corresponding to +1 and or +2 to allow initiation of spRNA synthesis at the −10/−35 promoter sequences.

We also compared reads from cells lacking σ^54^ to cells with the wild-type σ^54^ or its ΔRI form. Intriguingly, in several cases reads were higher when σ^54^ was absent, suggesting that the σ^54^RNAP holoenzyme can be repressive for some genes, either directly or indirectly. We inspected such genes for −12/−24 sequences and grouped them into four different classes based on the location of the σ^54^ binding motifs relative to the active −10/−35 promoter sequences (Figure 6 and Supplementary Table S3). In several cases (class I and II), we find that the σ^54^ recognition site is overlapping with the likely footprint of a σ^70^RNAP holoenzyme on these sites, suggesting a simple binding competition mechanism of repression by the σ^54^RNAP. Using purified components, we tested four σ^70^-regulated linear homoduplex promoters (*argT, chaC, mdfA* and *patA*) from class I and II for binding repression by σ^54^ *in vitro* (Figure 7 and Supplementary Figure S3). The σ^70^RNAP holoenzyme was allowed to generate small primed RNAs (spRNAs) using initiating dinucleotides (−1/+1 or +1/+2). The experiments were performed under two conditions. In the first, σ^54^ and σ^70^ were co-incubated with the RNAP prior to addition of promoter DNA probes. Here σ^54^ was in three-fold molar excess to σ^70^ and RNAP. This competition from excess σ^54^ reduced the amount of spRNA synthesised by 10–60% (Figure 7). In the second, σ^54^ was pre-incubated with the promoter DNA probes prior to addition of the σ^70^RNAP holoenzyme. Here, σ^70^ was in more than three-fold molar excess to σ^54^ and RNAP. Again, we observed a similar σ^54^-dependent reduction in spRNA synthesis (Supplementary Figure S3). Since the binding affinity and dissociation constant of σ^54^ and σ^70^ for RNAP are very similar (22) and similar results were obtained in both conditions, we suggest that inhibition is observed due to a binding competition from σ^54^ containing RNAP within the −10/−35 promoter sites rather than a direct competition for RNAP by σ^70^ and σ^54^.

In one other case the putative −12/−24 recognition site is far away from where the upregulated transcription start site is mapped to by RNAseq (Figure 6 class IV). Here, repression may occur not by simple competition for overlapping promoters, but for example through direct or indirect interferences between convergent transcribing RNA polymerases.

Interestingly, in contrast to bypass promoters (Table 1) the C −12 residue is present in all observed σ^54^ motifs correlating with local repression of transcription, consistent with the aforementioned repressive nature of GC at −12 (21).

### Cold-shock promoters have unusual σ^54^ dependencies

**Figure 8.**
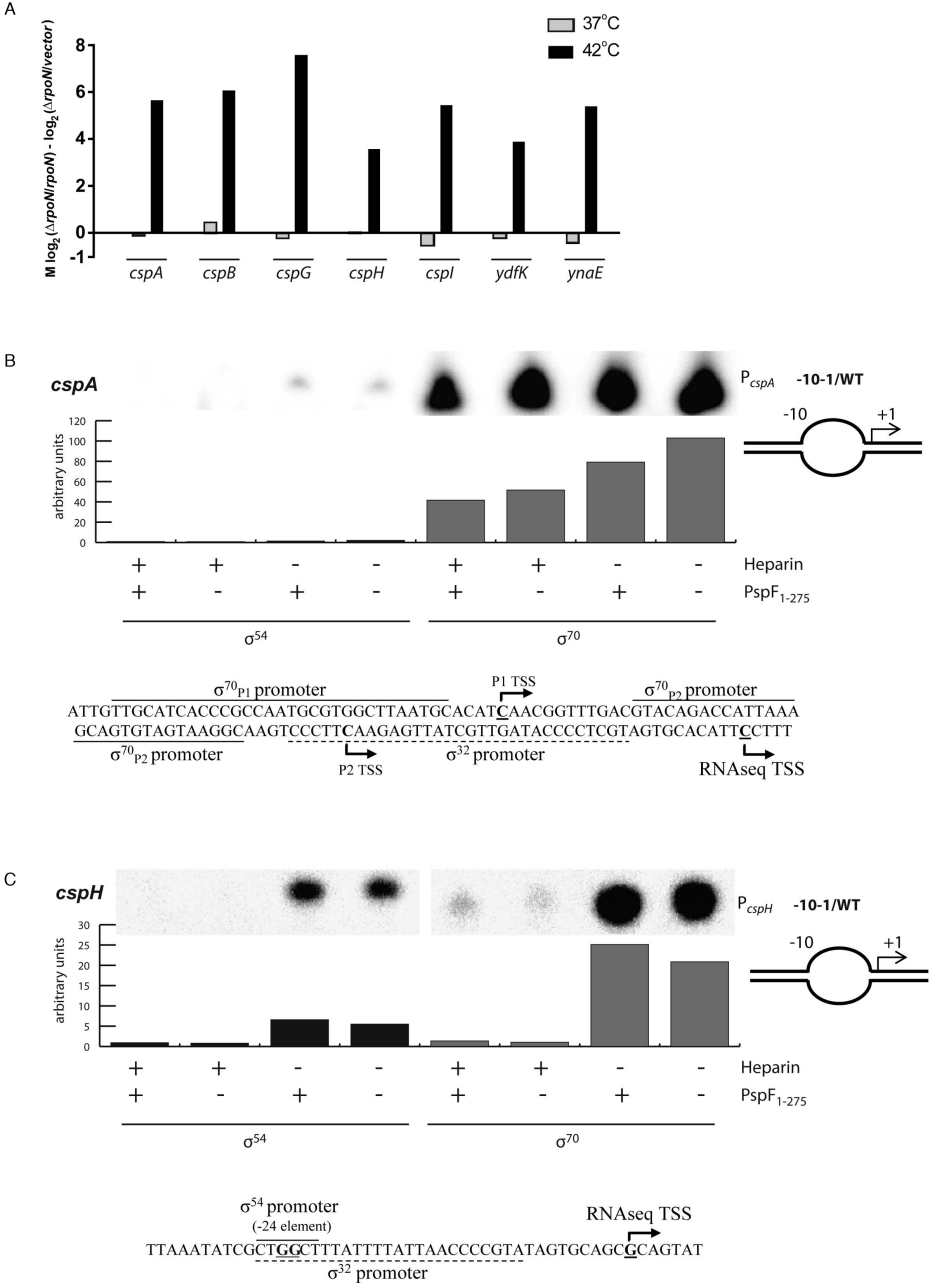
(**A**) Expression of cold-shock genes *in vivo* is upregulated in a σ^54^-dependent manner at 42°C but not at 37°C. *In vitro* transcription of *cspA* (**B**) and *cspH* (**C**) with the -10–1/WT heteroduplex promoters is σ^70^ dependent and largely σ^54^ independent.

Notably, some of the promoters dependent upon σ^54^ *in vivo* were driving the expression of cold-shock genes (Figure 8A). ChIP Seq identified a σ^54^ binding site in two of these genes (*cspA* and *cspH*) despite lacking any clear consensus −12/−24 sequences (Joe Wade, University of Albany, USA; personal communication). However, *in vitro* transcription from *cspA* (Figure 8B) and *cspH* (Figure 8C) promoters was found to be σ^70^-dependent and largely unresponsive to σ^54^. Thus, the basis of their σ^54^ dependence in *E. coli* remains unclear, however in *Bacillus subtilis* the σ^54^ homologue (σ^L^) has been shown to be essential for mounting a cold shock response through the transcription of σ^54^-dependent cold shock transcriptional regulators BkdR and YplP (23). Intriguingly, both *cspA* and *cspH* promoters contain putative heat shock sigma factor (σ^32^) binding motifs (24), consistent with the transcriptional start site mapped via the RNASeq reads (Figure 8B and C). Moreover, we only observe this σ^54^-dependent upregulation of cold shock genes at 42°C and not 37°C (Figure 8A). In all RNAseq experiments cells were cold harvested. The expression of cold shock promoters may therefore reflect the difference in the temperature shift from 42°C to ice and from 37°C to ice. We note however that σ^32^ activity has been suggested to be regulated by σ^54^ (25), raising the possibility that σ^54^ may be required for the expression of cold shock genes *cspA* and *cspH* by modulating the activity of σ^32^.

## DISCUSSION

The σ^54^-dependent gene expression paradigm is widely distributed amongst bacteria and is responsible for the expression of stress-induced genes important in many bacterial driven processes, especially the nitrogen cycle (26). Bioinformatics suggests the σ^54^ system is commonly associated with cell envelope function (27). Recent structural studies highlight how σ^54^ interacts with the core RNA polymerase to yield a holoenzyme unable to manipulate promoter DNA for making open promoter complexes until re-organised by its cognate AAA+ activator proteins (28). Studied here, the loss of the σ^54^ Region I from the holoenzyme frees up RNAP for promoter DNA delivery within and the subsequent templated synthesis of RNA (6,29–33). Our analysis of the properties of the bypass form of σ^54^ *in vivo* suggests such DNA delivery into the RNAP once Region I is missing is rarely occurring *in vivo*. *In vitro* the activator bypass phenotype of the ΔRI σ^54^ form is enhanced by using pre-opened DNA templates and conditions favouring DNA opening such as DNA supercoiling (6,32). The lack of an appropriate state of the promoter DNA available to the ΔRI form of the σ^54^RNAP holoenzyme may well be limiting for bypass transcription *in vivo*. The single gene KO enhancing bypass transcription encodes a nucleoid-associated protein (TtdR), and may influence DNA structure to normally repress bypass transcription.

Earlier work from the Gralla lab established that with single promoter studies (the *glnA*_p2_ promoter) bypass transcription could be elevated by altering the TGCA sequence around the −12 GC element (21). Where we did observe evidence for bypass transcription *in vivo* we noted a non-consensus −12 promoter sequence, the place where the Region I of σ^54^ interacts with DNA (34,35). We propose that the repressive interactions made by Region I may be compensated for by some promoter sequences associated with DNA delivery into RNAP. Indeed destroying the repressive fork junction structure formed near the −12 promoter element yields bypass transcription *in vitro* (6,21,36). Overall we conclude that to increase bypass transcription *in vivo* appropriate changes in local DNA structure favoring DNA opening and diminishing the repressive contributions of the −12 promoter sequences would need to occur. This view is congruent with the following observations: (i) correlation between low-level bypass transcription with a less conserved C −12 nucleotide and (ii) an apparent lack of *in trans* encoded repressors of bypass transcription.

Overlapping promoters and competition between sigma factors are recognized as being important regulatory features of a range of genes (37–41), although rather few cases are documented for σ^54^ (40). Here, we provide further evidence that, in addition to being a target of activators for regulated gene expression, σ^54^ can act to repress gene transcription by other forms of RNA polymerase through a binding competition for promoter sites. Our analysis of any repressive capacity is unlikely to be comprehensive, since a single growth condition was studied. Given more σ^54^ binding sites than conventional activator dependent σ^54^ promoters have been identified using a range of genomic methods, we propose that the repressive function is pervasive. Indeed, the σ^54^ factor is designed to inhibit RNAP by penetrating and blocking DNA entry routes (27,42,43), and it may well be that the earliest function of σ^54^ or some of its domains was to globally repress transcription. The unusual behaviours of the cold-shock promoters with respect to σ^54^ dependence is intriguing, and suggests some non-canonical functions of σ^54^ and or its holoenzyme remain to be discovered. These may involve factors outside of the well-recognized AAA+ activators which reorganize σ^54^ at its classical promoter sites.

## SUPPLEMENTARY DATA

Supplementary Data are available at NAR Online.

SUPPLEMENTARY DATA

## References

[1] Wigneshweraraj S., Bose D., Burrows P.C., Joly N., Schumacher J., Rappas M., Pape T., Zhang X., Stockley P., Severinov K. (2008). Modus operandi of the bacterial RNA polymerase containing the sigma54 promoter-specificity factor. Mol. Microbiol..

[2] Ghosh T., Bose D., Zhang X. (2010). Mechanisms for activating bacterial RNA polymerase. FEMS Microbiol. Rev..

[3] Rappas M., Bose D., Zhang X. (2007). Bacterial enhancer-binding proteins: sigma54-dependent gene transcription. Curr. Opin. Struct. Biol..

[4] Bush M., Dixon R. (2012). The role of bacterial enhancer binding proteins as specialized activators of sigma54-dependent transcription. Microbiol. Mol. Biol. Rev..

[5] Studholme D.J., Buck M. (2000). The biology of enhancer-dependent transcriptional regulation in bacteria: insights from genome sequences. FEMS Microbiol. Lett..

[6] Wang J.T., Syed A., Gralla J.D. (1997). Multiple pathways to bypass the enhancer requirement of sigma54 RNA polymerase: roles for DNA and protein determinants. Proc. Natl. Acad. Sci. U.S.A..

[7] Chaney M., Buck M. (1999). The sigma 54 DNA-binding domain includes a determinant of enhancer responsiveness. Mol. Microbiol..

[8] Engl C., Jovanovic G., Lloyd L.J., Murray H., Spitaler M., Ying L., Errington J., Buck M. (2009). *In vivo* localizations of membrane stress controllers PspA and PspG in *Escherichia coli*. Mol. Microbiol..

[9] Baba T., Ara T., Hasegawa M., Takai Y., Okumura Y., Baba M., Datsenko K.A., Tomita M., Wanner B.L., Mori H. (2006). Construction of Escherichia coli K-12 in-frame, single-gene knockout mutants: the Keio collection. Mol. Syst. Biol..

[10] Hobbs E.C., Astarita J.L., Storz G (2010). Small RNAs and small proteins involved in resistance to cell envelope stress and acid shock in *Escherichia coli*: analysis of a bar-coded mutant collection. J. Bacteriol..

[11] Gutnick D., Calvo J.M., Klopotowski T., Ames B.N. (1969). Compounds which serve as the sole source of carbon or nitrogen for *Salmonella typhimurium LT-2*. J. Bacteriol..

[12] Miller J.H. (1972). Experiments in Molecular Genetics.

[13] Mortazavi A., Williams B.A., McCue K., Schaeffer L., Wold B. (2008). Mapping and quantifying mammalian transcriptomes by RNA-Seq. Nat. Methods.

[14] Dago A.E., Wigneshweraraj S.R., Buck M., Morett E. (2007). A role for the conserved GAFTGA motif of AAA+ transcription activators in sensing promoter DNA conformation. J. Biol. Chem..

[15] Zhang N., Joly N., Burrows P.C., Jovanovic M., Wigneshweraraj S.R., Buck M. (2009). The role of the conserved phenylalanine in the sigma54-interacting GAFTGA motif of bacterial enhancer binding proteins. Nucleic Acids Res..

[16] Joly N., Engl C., Jovanovic G., Huvet M., Toni T., Sheng X., Stumpf M.P., Buck M. (2010). Managing membrane stress: the phage shock protein (Psp) response, from molecular mechanisms to physiology. FEMS Microbiol. Rev..

[17] Bergman J.M., Hammarlöf D.L., Hughes D. (2014). Reducing ppGpp level rescues an extreme growth defect caused by mutant EF-Tu. PLoS One.

[18] Barrios H., Valderrama B., Morett E. (1999). Compilation and analysis of sigma(54)-dependent promoter sequences. Nucleic Acids Res..

[19] Crooks G.E., Hon G., Chandonia J.M., Brenner S.E. (2004). WebLogo: a sequence logo generator. Genome Res..

[20] Schneider T.D., Stephens R.M. (1990). Sequence logos: a new way to display consensus sequences. Nucleic Acids Res..

[21] Wang L., Guo Y., Gralla J.D. (1999). Regulation of sigma 54-dependent transcription by core promoter sequences: role of −12 region nucleotides. J. Bacteriol..

[22] Maeda H., Fujita N., Ishihama A. (2000). Competition among seven *Escherichia coli* sigma subunits: relative binding affinities to the core RNA polymerase. Nucleic Acids Res..

[23] Wiegeshoff F., Beckering C.L., Debarbouille M., Marahiel M.A. (2006). Sigma L is important for cold shock adaptation of *Bacillus subtilis*. J. Bacteriol..

[24] Cowing D.W., Bardwell J.C., Craig E.A., Woolford C., Hendrix R.W., Gross C.A. (1985). Consensus sequence for Escherichia coli heat shock gene promoters. Proc. Natl. Acad. Sci. U.S.A..

[25] Pallen M. (1999). RpoN-dependent transcription of rpoH. Mol. Microbiol..

[26] Kustu S., Santero E., Keener J., Popham D., Weiss D. (1989). Expression of sigma 54 (ntrA)-dependent genes is probably united by a common mechanism. Microbiol. Rev..

[27] Francke C., Groot K.T., Hagemeijer Y., Overmars L., Sluijter V., Moezelaar R., Siezen R.J. (2011). Comparative analyses imply that the enigmatic Sigma factor 54 is a central controller of the bacterial exterior. BMC Genomics.

[28] Bose D., Pape T., Burrows P.C., Rappas M., Wigneshweraraj S.R., Buck M., Zhang X. (2008). Organization of an activator-bound RNA polymerase holoenzyme. Mol. Cell.

[29] Gallegos M.T., Cannon W.V., Buck M. (1999). Functions of the sigma(54) region I in trans and implications for transcription activation. J. Biol. Chem..

[30] Wang J.T., Syed A., Hsieh M., Gralla J.D. (1995). Converting Escherichia coli RNA polymerase into an enhancer-responsive enzyme: role of an NH2-terminal leucine patch in sigma 54. Science.

[31] Wang J.T., Gralla J.D. (1996). The transcription initiation pathway of sigma 54 mutants that bypass the enhancer protein requirement. Implications for the mechanism of activation. J. Biol. Chem..

[32] Cannon W., Chaney M., Buck M. (1999). Characterisation of holoenzyme lacking sigmaN regions I and II. Nucleic Acids Res..

[33] Syed A., Gralla J.D. (1998). Identification of an N-terminal region of sigma 54 required for enhancer responsiveness. J. Bacteriol..

[34] Chaney M., Buck M. (1999). The sigma 54 DNA-binding domain includes a determinant of enhancer responsiveness. Mol. Microbiol..

[35] Chaney M., Grande R., Wigneshweraraj S.R, Cannon W., Casaz P., Gallegos M.T., Schumacher J., Jones S., Elderkin S., Dago A.E. (2001). Binding of transcriptional activators to sigma 54 in the presence of the transition state analog ADP-aluminum fluoride: insights into activator mechanochemical action. Genes Dev..

[36] Guo Y., Wang L., Gralla J.D. (1999). A fork junction DNA-protein switch that controls promoter melting by the bacterial enhancer-dependent sigma factor. EMBO J..

[37] Wade J.T., Castro R.D., Grainger D.C., Hurd D., Busby S.J., Struhl K., Nudler E. (2006). Extensive functional overlap between sigma factors in *Escherichia coli*. Nat. Struct. Mol. Biol..

[38] Grainger D.C., Goldberg M.D., Lee D.J., Busby S.J. (2008). Selective repression by Fis and H-NS at the *Escherichia coli* dps promoter. Mol. Microbiol..

[39] Cho B.K., Kim D., Knight E.M., Zengler K., Palsson B.O. (2014). Genome-scale reconstitution of the sigma factor network in *Escherichia coli*: topology and functional states. BMC Biol..

[40] Zafar M.A., Carabetta V.J., Mandel M.J., Silhavy T.J. (2014). Transcriptional occlusion caused by overlapping promoters. Proc. Natl. Acad. Sci. U.S.A..

[41] Levi-Meyrueis C., Monteil V., Sismeiro O., Dillies M.A., Kolb A., Monot M., Dupuy B., Duarte S.S., Jagla B., Coppee J.Y. (2015). Repressor activity of the RpoS/σS-dependent RNA polymerase requires DNA binding. Nucleic Acids Res..

[42] Burrows P.C., Joly N., Buck M. (2010). A prehydrolysis state of an AAA +ATPase supports transcription activation of an enhancer-dependent RNA polymerase. Proc. Natl. Acad. Sci. U.S.A..

[43] Sharma A., Leach R.N., Gell C., Zhang N., Burrows P.C., Shepherd D.A., Wigneshweraraj S., Smith D.A., Zhang X., Buck M. (2014). Domain movements of the enhancer-dependent sigma factor drive DNA delivery into the RNA polymerase active site: insights from single molecule studies. Nucleic Acids Res..

