# Supplementary material for: Genome wide interactions of wild-type and activator bypass forms of σ54
Source: Nucleic Acids Res. 2015 Jun 16;43(15):7280–91. doi: 10.1093/nar/gkv597 (PMC4551910; doi:10.1093/nar/gkv597)
Supplement: SUPPLEMENTARY DATA [file supp_43_15_7280__index.html]

Genome wide interactions of wild-type and activator bypass forms of σ54 — SUPPLEMENTARY DATA 

# Genome wide interactions of wild-type and activator bypass forms of σ54

## SUPPLEMENTARY DATA

- SUPPLEMENTARY DATA
- SUPPLEMENTARY DATA
