# Supplementary material for: Genome wide interactions of wild-type and activator bypass forms of σ54
Source: Nucleic Acids Res. 2015 Jun 16;43(15):7280–91. doi: 10.1093/nar/gkv597 (PMC4551910; doi:10.1093/nar/gkv597)
Supplement: SUPPLEMENTARY DATA [file supp_gkv597_nar-01035-v-2015-File013.pdf]

SUPPLEMENTARY DATA

Table S1

Strain	Genetic background
KEIO pool (Gift from C. Gross,UCSF)	In-frame single-gene substitution of 3985 genes in BW25113 (Baba *et al.*, 2006).
BW25113	*rrnB3 ΔlacZ4787 hsdR514 Δ(araBAD)567 Δ(rhaBAD)568 rph-1*
K1471 (from CGSC, Yale)	F-, *Δ(araA-leu)7697*, *[araD139]*$_{B/r}$, *Δ(codB-lacI)3*, *galK16*, *galE15*(GalS), λ^-, *e14-*, *mcrA0*, *relA1*, ***rpoN208::Tn10***, *rpsL150*(strR), *spoT1*, *mcrB1*, *hsdR2*
MVA4 (Gift from G. Jovanovic)	MC1061 **ΦP$_{pspA}$-*lacZ*** (Amp^R)
MG1655	F- λ- *ilvG- rfb-50 rph-1*
Small peptide/small RNA library (Gift from G. Storz, NIH)	Bar-coded in-frame single-gene substitution of 125 genes encoding small peptides / small RNAs in MG1655 (Hobbs *et al.*, 2010)
gln$_{Ap2}$-WTRS	BW25113, *rpoN208::Tn10*, pSB4A3- ΦP$_{glnAp2}$-*gfp*, pBAD18*cm(rpoN)*
gln$_{AP2}$-SABRS	BW25113, *rpoN208::Tn10*, pSB4A3- ΦP$_{glnAp2}$-*gfp*, pBAD18cm(*rpoN*$_{\Delta R1}$)
gln$_{Ap2}$-DRRS	BW25113, *rpoN208::Tn10*, pSB4A3- ΦP$_{glnAp2}$-*gfp*, pBAD18cm
pspA-WTRS	BW25113, *rpoN208::Tn10*, ΦP$_{pspA}$-*lacZ*, pBAD18cm*(rpoN)*
pspA-SABRS	BW25113, *rpoN208::Tn10*, ΦP$_{pspA}$-*lacZ*, pBAD18cm(*rpoN*$_{\Delta R1}$)
pspA-DRRS	BW25113, *rpoN208::Tn10*, ΦP$_{pspA}$-*lacZ*, pBAD18cm

Table S1: Shown are the strains used in this study and their relevant genetic traits. Wild type *rpoN* strain (WTRS), σ^{54} activator bypass *rpoN* strain (SABRS), deletion *rpoN* strain (DRRS).

Table S2

	Genetic Background		XGal	MacConkey	Temperature	0.001% arabinose	Miller Units [%]	Gene ID
Δ*rpoN*	*pBAD18cm*		-	-	37°C	-	4	
Δ*rpoN*	*pBAD18cm(rpoN)*		++	++	37°C	-	100	
Δ*rpoN*	*pBAD18cm(rpoN*$_{\Delta RI}$*)*		-	-	37°C	-	3	
Δ*rpoN*	*pBAD18cm(rpoN*$_{\Delta RI}$*)*	KO1	+	+	37°C	-	56	*hldE*
Δ*rpoN*	*pBAD18cm(rpoN*$_{\Delta RI}$*)*	KO2	+	+	37°C	-	21	*yhbX*
Δ*rpoN*	*pBAD18cm(rpoN*$_{\Delta RI}$*)*	KO3	+	+	37°C	-	8	
Δ*rpoN*	*pBAD18cm(rpoN*$_{\Delta RI}$*)*	KO4	+	+	37°C	-	9	
Δ*rpoN*	*pBAD18cm(rpoN*$_{\Delta RI}$*)*	KO5	+	+	37°C	-	52	*nanT*
Δ*rpoN*	*pBAD18cm(rpoN*$_{\Delta RI}$*)*	KO6	+	+	37°C	-	11	*ttdR*
Δ*rpoN*	*pBAD18cm(rpoN*$_{\Delta RI}$*)*	KO7	+	+	37°C	-	13	*asnA*
Δ*rpoN*	*pBAD18cm(rpoN*$_{\Delta RI}$*)*	KO8	+	+	37°C	-	26	*greA*
Δ*rpoN*	*pBAD18cm*		-	-	37°C	+	5	
Δ*rpoN*	*pBAD18cm(rpoN)*		++	++	37°C	+	100	
Δ*rpoN*	*pBAD18cm(rpoN*$_{\Delta RI}$*)*		-	-	37°C	+	2	
Δ*rpoN*	*pBAD18cm(rpoN*$_{\Delta RI}$*)*	KO9	+	+	37°C	+	7	
Δ*rpoN*	*pBAD18cm(rpoN*$_{\Delta RI}$*)*	KO10	+	+	37°C	+	1	
Δ*rpoN*	*pBAD18cm(rpoN*$_{\Delta RI}$*)*	KO11	+	+	37°C	+	0	
Δ*rpoN*	*pBAD18cm(rpoN*$_{\Delta RI}$*)*	KO12	+	+	37°C	+	0	
Δ*rpoN*	*pBAD18cm(rpoN*$_{\Delta RI}$*)*	KO13	+	+	37°C	+	5	
Δ*rpoN*	*pBAD18cm(rpoN*$_{\Delta RI}$*)*	KO14	+	+	37°C	+	0	
Δ*rpoN*	*pBAD18cm(rpoN*$_{\Delta RI}$*)*	KO15	+	+	37°C	+	6	
Δ*rpoN*	*pBAD18cm(rpoN*$_{\Delta RI}$*)*	KO16	+	+	37°C	+	4	
Δ*rpoN*	*pBAD18cm(rpoN*$_{\Delta RI}$*)*	KO17	+	+	37°C	+	2	
Δ*rpoN*	*pBAD18cm(rpoN*$_{\Delta RI}$*)*	KO18	+	+	37°C	+	0	
Δ*rpoN*	*pBAD18cm*		-	-	42°C	-	11	
Δ*rpoN*	*pBAD18cm(rpoN)*		++	++	42°C	-	100	
Δ*rpoN*	*pBAD18cm(rpoN*$_{\Delta RI}$*)*		-	-	42°C	-	7	
Δ*rpoN*	*pBAD18cm(rpoN*$_{\Delta RI}$*)*	KO19	+	+	42°C	-	16	
Δ*rpoN*	*pBAD18cm(rpoN*$_{\Delta RI}$*)*	KO20	+	+	42°C	-	7	
Δ*rpoN*	*pBAD18cm(rpoN*$_{\Delta RI}$*)*	KO21	+	+	42°C	-	5	
Δ*rpoN*	*pBAD18cm(rpoN*$_{\Delta RI}$*)*	KO22	+	+	42°C	-	7	
Δ*rpoN*	*pBAD18cm(rpoN*$_{\Delta RI}$*)*	KO23	+	+	42°C	-	9	
Δ*rpoN*	*pBAD18cm(rpoN*$_{\Delta RI}$*)*	KO24	+	+	42°C	-	12	
Δ*rpoN*	*pBAD18cm(rpoN*$_{\Delta RI}$*)*	KO25	+	+	42°C	-	6	
Δ*rpoN*	*pBAD18cm(rpoN*$_{\Delta RI}$*)*	KO26	+	+	42°C	-	10	
Δ*rpoN*	*pBAD18cm*		-	-	37°C	-	4	
Δ*rpoN*	*pBAD18cm(rpoN)*		++	++	37°C	-	100	
Δ*rpoN*	*pBAD18cm(rpoN*$_{\Delta RI}$*)*		-	-	37°C	-	5	
Δ*rpoN*	*pBAD18cm(rpoN*$_{\Delta RI}$*)*	ST 1	+	+	37°C	-	3	
Δ*rpoN*	*pBAD18cm(rpoN*$_{\Delta RI}$*)*	ST 2	+	+	37°C	-	1	
Δ*rpoN*	*pBAD18cm(rpoN*$_{\Delta RI}$*)*	ST 3	+	+	37°C	-	0	
Δ*rpoN*	*pBAD18cm(rpoN*$_{\Delta RI}$*)*	ST 4	+	+	37°C	-	0	
Δ*rpoN*	*pBAD18cm(rpoN*$_{\Delta RI}$*)*	ST 5	+	+	37°C	-	2	
Δ*rpoN*	*pBAD18cm(rpoN*$_{\Delta RI}$*)*	ST 6	+	+	37°C	-	4	
Δ*rpoN*	*pBAD18cm(rpoN*$_{\Delta RI}$*)*	ST 7	+	+	37°C	-	9	
Δ*rpoN*	*pBAD18cm(rpoN*$_{\Delta RI}$*)*	ST 8	+	+	37°C	-	3	
Δ*rpoN*	*pBAD18cm(rpoN*$_{\Delta RI}$*)*	ST 9	+	+	37°C	-	0	
Δ*rpoN*	*pBAD18cm(rpoN*$_{\Delta RI}$*)*	ST 10	+	+	37°C	-	4	

Table S2: ΦP$_{pspA}$-*lacZ* activity of selected single-gene KOs in the *pspA*-SABRS measured at 37°C, 42°C, and 0.001% arabinose (using XGal/MacConkey indicator plates and β-galactosidase assays). Also shown are the activities of overexpression clones (ST1-10) obtained using a *Salmonella typhimurium LT2* library (a gift from Diarmaid Hughes, 16).

Table S3

Class I binding motifs: Overlapping with promoter

Gene	Expression (*rpoN*/ vector)	Binding motif	Gene length	Promoter Sequence
chaC	29%	-10 to -26	696	G**TGG**TCCGTGGA**TTGC**A
patA	48%	-9 to -25	1,380	G**TGGC**GCAATCCC**TGC**A
ybhK	79%	-22 to -38	909	T**TGGC**AGGTTAA**TTGC**T

Class II binding motifs: Downstream of promoter

Gene	Expression (*rpoN*/ vector)	Binding motif	Gene length	Promoter Sequence
argT	23%	+6 to +22	783	A**TGGC**ATAAGACC**TGC**A
mdfA	43%	+11 to +27	1,233	T**TGGC**GAAGAAA**TTGC**A
topA	59%	-9 to +7	2,598	C**TGGC**AATAGAT**TTGC**T

Class III binding motifs: intragenic

Gene	Expression (*rpoN*/ vector)	Binding motif	Gene length	Promoter Sequence
argS	59%	597-613	1,834	TC**GG**TATGCTGA**TTGC**A
arnT	68%	1,487-1,503	1,753	C**TGGC**ATGGAGCC**TGC**A
bioB	21%	437-453	1,141	C**TGG**AACAAATGG**TGC**A
carB	12%	2,546-2,562	3,332	C**TGGC**CTTCGAA**TTGC**A
evgS	15%	2,098-2,114	3,694	C**TGGC**ATACATTA**TGC**A
fimH	46%	528-544	1,003	C**TGGC**TCATTAA**TTGC**C
glgA	64%	597-613	1,534	AA**GGC**ATGTTTTA**TGC**A
groL	43%	1,413-1,419	1,747	TG**GG**TATCAAAG**TTGC**A
holD	57%	463-479	514	A**TGGC**AACAAAT**TTGC**A
nagK	66%	123-139	1,012	G**TGG**AACAAAAA**TTGC**G
narY	46%	1,112-1,128	1,645	C**TGGC**ACTACCG**TTGC**A
ptrA	56%	2,246-2,262	2,989	C**TGGC**ACGCGATG**TGC**A
purK	6%	815-831	1,168	C**TGGC**ACCGCGTG**TGC**A
qseC	52%	1,166-1,182	1,450	T**TGG**TGCGAAAT**TTGC**T
uxaC	67%	948-964	1,513	T**TGG**TGCCGAATA**TGC**A
wbbK	46%	807-823	1,219	G**TGG**TACAGAAAA**TGC**G
ygiB	64%	510-526	772	GC**GGC**GCGGGAT**TTGC**A
yjjV	65%	695-711	880	C**TGGC**ATCGTTA**TTGC**T
yncD	36%	2,172-2,188	2,203	TC**GGC**ATGAATA**TTGC**G

Class IV binding motifs: intergenic antisense

Gene	Expression (*rpoN*/ vector)	Binding motif	Gene length	Promoter Sequence
katE	15%	2600-2584	2,362	GC**GGC**GTGAACC**TTGC**A

Table S3: List of genes with σ^{54} ChIP Seq binding sites (Joe Wade, personal communication) repressed in the presence of RpoN. Genes are grouped into 4 classes according to the position of the σ^{54} binding motif relative to the start site of the σ^{70} like promoter showing σ^{54} sensitivity. Class I binding motifs overlap with the σ^{70}-like promoter, Class II motifs are located downstream of the promoter sequence and upstream of the translation start site, Class III motifs are intragenic, and Class IV motifs are downstream of the gene in the antisense direction. The location of the σ^{54} binding motifs relative to the transcription start site of the σ^{70}-like promoter is also shown.

Table S4: RNA sequencing reads aligned to the NC_000913 *E. coli* K-12 reference genome and statistical analysis.

Figure S1

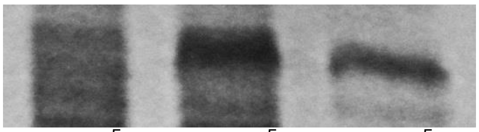

Supplementary figure S1: Expression of σ^{54} and $\sigma^{54}_{\Delta R1}$ confirmed via SDS-PAGE, and by RNA Sequencing.

Figure S2

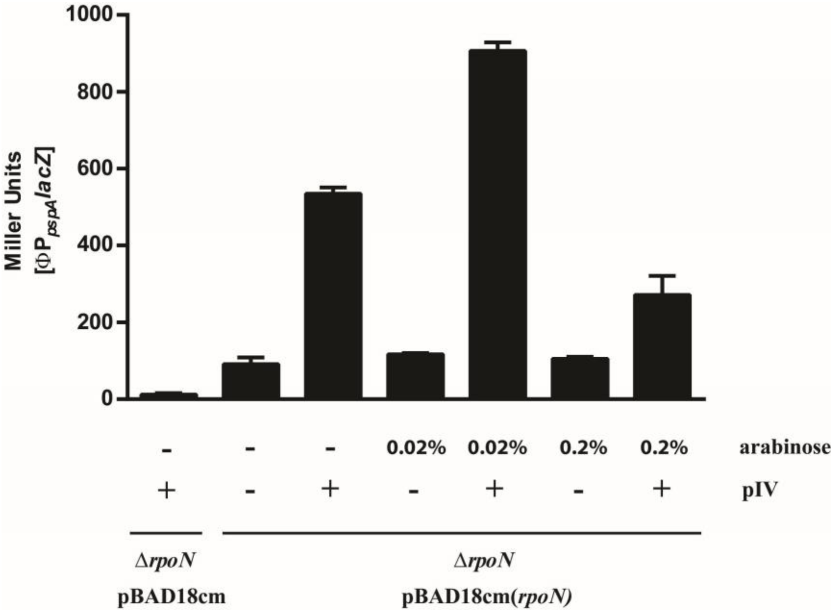

Supplementary figure S2: Leaky expression (no arabinose) of RpoN from pBAD18cm is sufficient for pIV-dependent induction of P_{pspA}-*lacZ*. RpoN is expressed from pBAD18cm under the control of the arabinose inducible P_{araBAD} promoter. Shown is the activity of the σ^{54}-dependent pIV inducible P_{pspA}-*lacZ* reporter in absence and in presence of 0.02% and 0.2% arabinose.

Figure S3

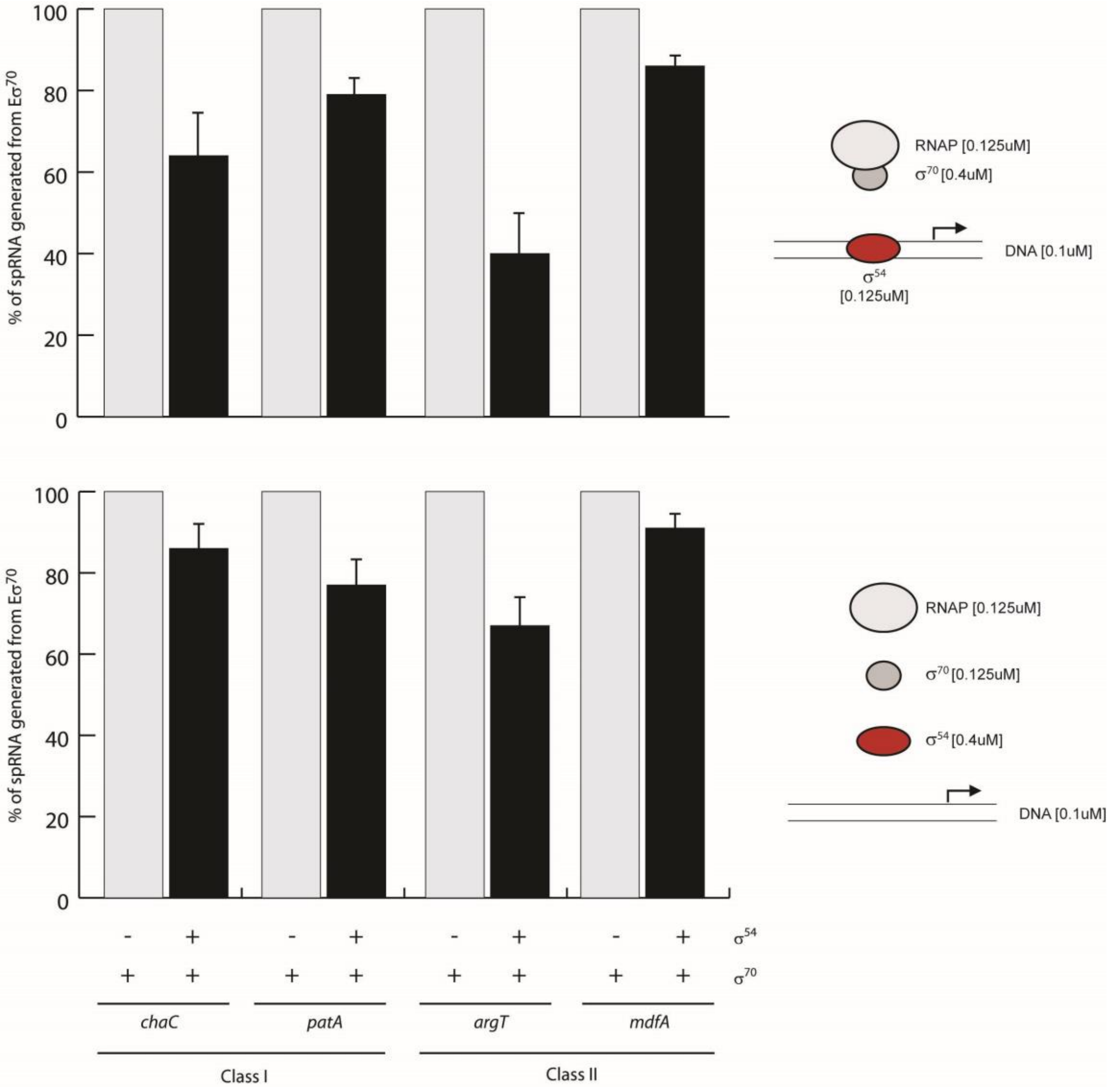

Supplementary figure S3: Four σ^{70}-regulated linear homoduplex promoters (*argT, chaC, mdfA* and *patA*) from classes I and II were tested for their promoter-binding repression by σ^{54} *in vitro* using purified components. **Top:** σ^{54} was pre-incubated with the promoter DNA probes prior to addition of the σ^{70}RNAP holoenzyme. Here, σ^{70} was in more than 3-fold molar excess to σ^{54} and RNAP. **Bottom:** σ^{54} and σ^{70} were co-incubated with the RNAP prior to addition of promoter DNA probes. Here σ^{54} was in 3-fold molar excess to σ^{70} and RNAP. A similar σ^{54}-dependent reduction in spRNA synthesis was observed across these experiments. The cartoons on the right illustrate the order of addition as well as the final concentrations of the individual components.
